# Enhanced Production of Nitrogenated Metabolites with Anticancer Potential in *Aristolochia manshuriensis* Hairy Root Cultures

**DOI:** 10.3390/ijms241411240

**Published:** 2023-07-08

**Authors:** Yury N. Shkryl, Galina K. Tchernoded, Yulia A. Yugay, Valeria P. Grigorchuk, Maria R. Sorokina, Tatiana Y. Gorpenchenko, Olesya D. Kudinova, Anton I. Degtyarenko, Maria S. Onishchenko, Nikita A. Shved, Vadim V. Kumeiko, Victor P. Bulgakov

**Affiliations:** 1 Federal Scientific Center of the East Asia Terrestrial Biodiversity of the Far East Branch of Russian Academy of Sciences, 159 Stoletija Str., 690022 Vladivostok, Russiadegtyarenko@biosoil.ru (A.I.D.);; 2 Department of Medical Biology and Biotechnology, Far Eastern Federal University, 690950 Vladivostok, Russia; 3 A.V. Zhirmunsky National Scientific Center of Marine Biology, Far Eastern Branch of the Russian Academy of Sciences, 690041 Vladivostok, Russia

**Keywords:** *rolB*, *rolC*, Mu Tong, magnoflorine, aristolochic acids, anticancer

## Abstract

*Aristolochia manshuriensis* is a relic liana, which is widely used in traditional Chinese herbal medicine and is endemic to the Manchurian floristic region. Since this plant is rare and slow-growing, alternative sources of its valuable compounds could be explored. Herein, we established hairy root cultures of *A. manshuriensis* transformed with *Agrobacterium rhizogenes* root oncogenic loci (*rol)B* and *rolC* genes. The accumulation of nitrogenous secondary metabolites significantly improved in transgenic cell cultures. Specifically, the production of magnoflorine reached up to 5.72 mg/g of dry weight, which is 5.8 times higher than the control calli and 1.7 times higher than in wild-growing liana. Simultaneously, the amounts of aristolochic acids I and II, responsible for the toxicity of *Aristolochia* species, decreased by more than 10 fold. Consequently, the hairy root extracts demonstrated pronounced cytotoxicity against human glioblastoma cells (U-87 MG), cervical cancer cells (HeLa CCL-2), and colon carcinoma (RKO) cells. However, they did not exhibit significant activity against triple-negative breast cancer cells (MDA-MB-231). Our findings suggest that hairy root cultures of *A. manshuriensis* could be considered for the rational production of valuable *A. manshuriensis* compounds by the modification of secondary metabolism.

## 1. Introduction

*Aristolochia* L. belongs to a large genus of Aristolochiaceae, consisting of about 500 species, mainly distributed in tropical and subtropical regions [1]. Extensive research has been conducted on the unique pollination mechanisms employed by *Aristolochia* species, which involve a floral aroma and a perianth that entraps insects. Additionally, these species exhibit a species-specific relationship as host plants that serve as a habitat for swallowtail butterfly larvae [2,3,4]. The *Aristolochia* genus has garnered significant interest as a source of naturally occurring compounds with potential pharmaceutical properties. These compounds include nitrogen-containing secondary metabolites, flavonoids, lignans, terpenoids, and steroids. Consequently, *Aristolochia* plants are considered “natural factories” for producing these phytochemicals [5]. For centuries, *Aristolochia* species have been utilized in traditional medicine across Asia to treat various ailments, such as arthritis, rheumatism, angina, and myocardial infarction, alone or in combination with other herbs [6]. Studies have also suggested their potential as analgesics, antimalarials, antibacterial agents, and antidotes for snake bites [6,7]. However, certain *Aristolochia* species have been associated with an elevated risk of urinary tract cancer and renal interstitial fibrosis [7,8,9]. Consequently, their use in herbal medicinal products or dietary supplements has been banned [7,8].

The relict plant *A. manshuriensis* Kom. is an endemic of the Manchurian floristic region, native to Korea, China, and the southeastern part of Russia [10]. The dried stems of *A. manshuriensis*, known as Guan Mu Tong, are extensively used in traditional Chinese medicine [11]. Aristolochic acids (AAs) I, II, IIIa, IV, aristoloside, aristolamides, aristolactams, and alkaloids such as magnoflorine, manshurienine, tetrandrine, and aristopyridinone have been identified as the primary chemical constituents of *A. manshuriensis* [12,13,14]. These compounds are derived from L-tyrosine via (S)-norcoclaurine, with (S)-reticuline serving as the pivotal intermediate in the biosynthetic pathway for both phenanthrene derivatives and alkaloids [15]. Though *A. manshuriensis* plants contain AAs that cause nephrotoxicity and mutagenic and carcinogenic effects after prolonged use [16,17,18], it also accumulates various substances with excellent therapeutic potential. Aristolactam-type compounds from *A. manshuriensis* possess anti-tumor potential as inhibitors of cyclin-dependent kinase 2 [19]. Aristolamide II isolated from stems of *A. manshuriensis* showed an anti-inflammatory effect against superoxide anion production and human neutrophils releasing elastase. At the same time, AAs had no impact on either of the inflammatory mediators [13]. The polar quaternary aporphine magnoflorine is an important alkaloid with growing interest because of its broad spectrum of pharmacological properties, including anti-diabetic, anti-inflammatory, neuropsychopharmacological, immunomodulatory, hypotensive, antioxidant, and antifungal activities [20]. Magnoflorine reduced ischemia-induced neuronal damage in the cerebral cortex of rats, possibly accompanied by antioxidative stress, autophagy suppression, and Sirt1/AMPK pathway activation [21]. For practical purposes, it is crucial to obtain *A. manshuriensis* plant material containing high levels of beneficial compounds while minimizing harmful ones.

Propagation of *Aristolochia* is conducted through vegetative plant parts, seeds, or in vitro regeneration. These processes are time-consuming and labor-intensive. Though in vitro regeneration methods have been developed for *A. indica* [22], *A. cathcartii* [23], and *A. tagala* [24], pollination and fertilization in these plants cause weak fruit sets. Other limitations also include a small population, anthropogenic factors, and the absence of seed-dispersing agents [25,26]. Moreover, *A. manshuriensis* populations are affected by genetic drift due to a decline in the reproductive and effective population sizes, mainly linked to anthropogenic factors [27]. Therefore, developing an alternative plant biomass source is an exceedingly urgent task. In this regard, plant tissue culture techniques could become a potential tool for producing secondary metabolites of *A. manshuriensis*.

Plant cell cultures techniques, such as callus and cell suspension, have emerged as potential tools for producing secondary metabolites from medicinal plants. They can be used for the industrial and commercial production of essential plant chemicals because of their rapid growth and better productivity [28]. In vitro cultivation is an effective method for preserving rare and endangered natural species and protecting biodiversity. For example, callus cultures derived from leaf explants of *A. indica* and *A. bracteolata* have been shown to produce AA-I and AA-II [29]. Similarly, callus cultures of *A. tuberosa* have been found to produce 1.8 times more AAs than the original plant organs [30]. In addition, genetic transformation has been used to modify secondary metabolism and rationally produce valuable *Aristolochia* compounds. For instance, a suspension culture of *A. manshuriensis* transformed by wild-type *Agrobacterium tumefaciens* strain C58 with cardiotonic activity lost its ability to produce AAs [31]. Hairy roots, induced by the soil bacterium *A. rhizogenes*, are another promising approach for obtaining secondary metabolites, as they are fast-growing and higher-yielding compared to cell cultures [32,33]. However, there have been no studies on the development of hairy roots from *Aristolochia* species.

The present study establishes hairy root cultures of *A. manshuriensis* and investigates the effect of the *rolB* and *rolC* gene transfer on the metabolite profile of transformed cells. The anti-radical potential of the extracts and their cytotoxicity are also investigated.

## 2. Results and Discussion

### 2.1. Effect of Explants on Hairy Root Induction

**Figure 1:**
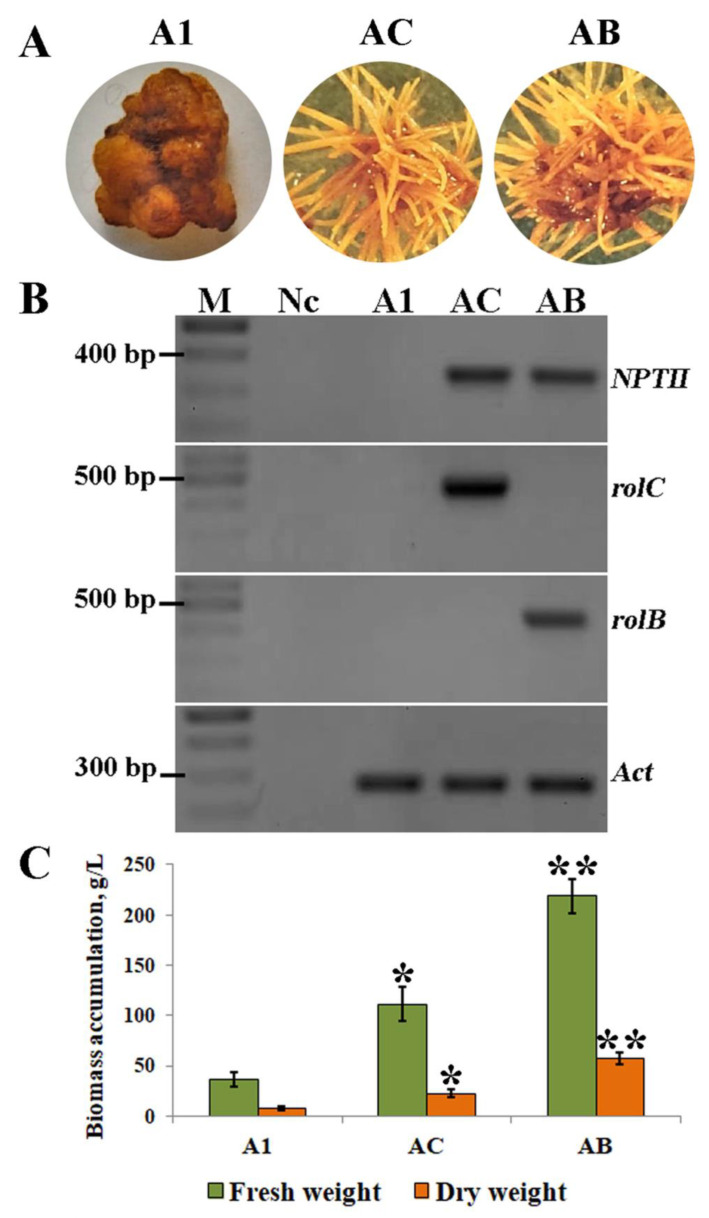
(**A**) Phenotypes of the *A. manshuriensis* cell cultures. A1—untransformed callus line; AC—hairy root culture transformed with the *rolC* gene; AB—hairy root culture transformed with the *rolB* gene. (**B**) PCR products of *nptII*, *rolC*, *rolB*, and *actin* from the genomic DNA of A1, AC, and AB cell lines. M—DNA markers (100 bp + 1.5 kb ladder, SybEnzyme, Russia); Nc—negative control (no DNA added). The original image is shown in Figure S2. (**C**) Biomass accumulation in 4-week-old callus and hairy root cultures of *A. manshuriensis*. Data are presented as the mean values ± standard errors, * *p* < 0.05, ** *p* < 0.01 (Student’s *t*-test).

Explants from the leaves, petioles, and stems of *A. manshuriensis* plants were infected with *A. tumefaciens* harboring *rolA*, *rolB*, and *rolC* genes to induce hairy root formation. They were cultured in a medium without hormones. Two weeks after infection, primary tumors were observed to be developing on *rol*-infected explants. After another week, adventitious roots emerged in the *rolC*- and *rolB*-infected stem and petiole-derived tumors, while no root growth was observed in *rolA*-transformed tumors. The induction of hairy roots in petiole explants infected with *A. tumefaciens* was lower compared to stems, while no induction effects were observed in the leaves (Table 1). In addition, it was observed that the development of adventitious roots was 2 times higher in stem and 1.3 times higher in petiole-type tumors infected with the *rolB* gene than those with the *rolC* gene (Table 1). The *rolA* calli grew very slowly as compact yellow globular aggregates, could not form roots, and were not included in further analysis. The control explants infected with *A. tumefaciens* bearing empty vectors (without *rol* genes) also showed no visible signs of root induction. Adventitious roots growing on stem-derived tumors were excised and transferred into the liquid media resulting in AC and AB hairy root cultures for the *rolC* and *rolB* transgenes, respectively. AC and AB cultures had yellow color and extensive lateral branching, typical for hairy root cultures (Figure 1A). The untransformed callus culture (A1) was previously derived from *A. manshuriensis* stems [34] and was taken as a control in this study.

To prove the integration of the T-DNA region of pPCV002 plasmid into the hairy root cultures of *A. manshuriensis*, PCR analysis of the *rol* and *nptII* genes was performed. Using genomic DNA from A1, AB, and AC cell lines, fragments corresponding to *nptII*, *rolC*, and *rolB* with the predicted lengths were amplified, which confirmed the successful genetic transformation in AC and AB cultures (Figure 1B).

The accumulation of fresh and dry biomass of *A. manshuriensis* cell cultures was observed for five weeks. The exponential phase of the growth curve for all lines was observed from the third to the fourth week when a noticeably faster rate of biomass accumulation was detected (Figure S1). Comparatively, the four-week cultures showed the highest fresh and dry weights at 36.6 and 7.4 g/L for A1, 111.4 and 22.5 g/L for AC, and 219.0 and 57.5 g/L for AB, respectively (Figure 1C).

The efficacy of hairy root syndrome induction is influenced by various factors, including but not limited to the type of explant, co-cultivation conditions, the concentration of acetosyringone, and *Agrobacterium* strain, each exerting varying degrees of control [35,36]. In this investigation, previously optimized transformation conditions [37] were taken advantage of, and the effectiveness of hairy root induction on different types of explants by different *rol* genes was studied. Consistent with our findings, higher transformation rates from stem explants have also been documented in *Trigonella foenum-graecum* [38], *Artemisia pallens* [39], and *Agastache rugosa* [40]. Petiole explants are effective for inducing hairy roots in *Withania somnifera* [41], *Isatis tinctoria* [42], and *Saussurea medusa* [43]. In contrast, for many plant species, leaf explants have exhibited better performance [35,44,45,46]. This suggests that the availability of cells competent to serve as root initials in different types of explants can play a critical role in the successful induction of hairy roots. Additionally, it is essential to note that different *Aristolochia* species may have unique requirements for successful transformation. The results obtained for *A. manshuriensis* may not necessarily apply to other species. Therefore, a systematic approach should be taken to optimize transformation protocols for each species of interest.

As for individual *rol* genes, the higher root-inducing capacity of *rolB* is not surprising. Although all three loci (i.e., *rolA*, *B*, and *C*) could induce hairy root syndrome in tobacco, only the *rolB* gene induced root formation in *Kalanchoe* [47]. A rhizogenic effect was not observed for the *rolA* gene in either carrot discs or ginseng cell transformation [48,49]. Moreover, the rhizogenic effect of the *rolA* gene in tobacco was weaker than that of the *rolC* [47]. It is supposed that the RolB protein possesses the highest morphogenetic activity among the *Agrobacterium* oncogenes [50,51]. At the same time, RolC supports the growth potential by counteracting the negative effects of RolB (necrosis and cell death) [52,53]. The capacity of wild-type *A. rhizogenes* to trigger hairy roots on carrot disks has been considerably diminished by CRISPR/Cas9-mediated mutations in *rolC* and *rolB* genes [54]. Interestingly, according to our previous results, the *rolC* gene stimulates cell growth, while the *rolB* gene considerably suppresses biomass accumulation, especially at high levels of transgene expression [55]. Conversely, in *A. manshuriensis* hairy roots, the *rolB* gene provided better growth properties than *rolC*. AC and AB cell cultures grow much faster than the A1 callus line. Analogous to our results, hairy roots of many different plant species demonstrate superior growth potency over calli cells [32,33].

### 2.2. The Effect of rolC and rolB on Accumulation of Phenanthroic Acid Derivatives in Hairy Root Cultures of A. manshuriensis

**Figure 2:**
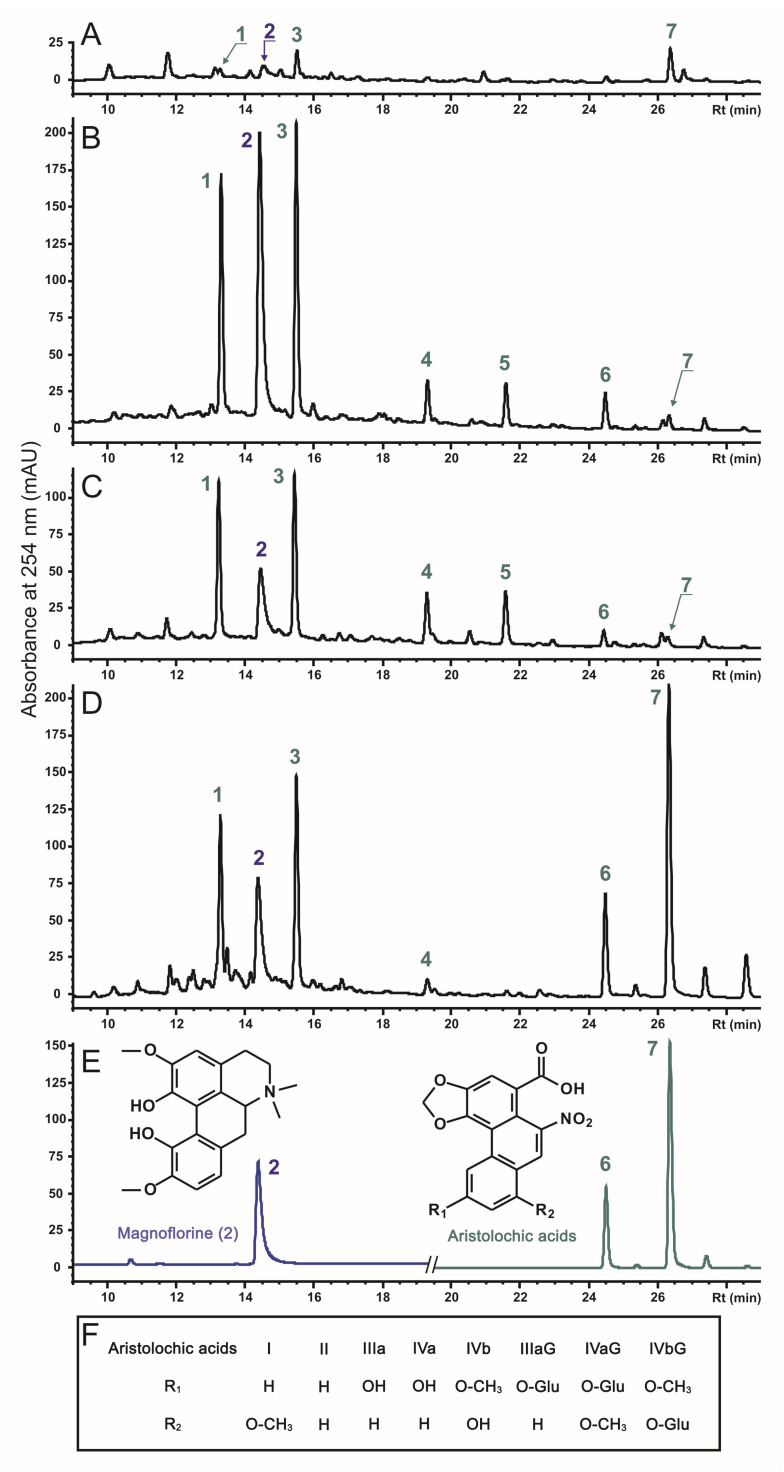
The HPLC-UV separation of main phenanthrene derivatives from (**A**) control cell line A1, (**B**) *rolC*-transgenic cell line AC, (**C**) *rolB*-transgenic cell line AB, and (**D**) stems of *A. manshuriensis* recorded at 254 nm. Identified compounds: aristolochic acid IIIa *O*-glucoside (1), magnoflorine (2), aristolochic acid IVa/IVb *O*-glucoside (3), aristolochic acid IIIa (4), aristolochic acid IVa/IVb (5), aristolochic acids II (6), aristolochic acids I (7). Representative chromatogram of standard compounds and chemical structures of the identified phenanthrene derivatives are also shown (**E**,**F**).

Through HPLC-UV-MS analysis of the control culture A1, hairy roots (AC and AB), and stems of *A. manshuriensis*, seven main phenanthroic acid derivatives were identified, including six aristolochic acids (AAs) and one aporphine alkaloid (Figure 2). All compounds were identified by comparing their UV and MS data, including retention time, UV maxima, accurate mass, and fragmentation pattern, with those of the available standards and literature data [56,57,58]. The informative data obtained from the analysis are summarized in Table S1 and Supplementary HPLC-UV and HRMS data. Magnoflorine (2), an aporphine derivative, and two aristolochic acids (AA)-I (7) and AA-II (6) were identified by complete concurrence with standard compounds. The mass spectrometric behavior of AAs was studied using standard compounds (AA-I and AA-II) and previously published information [56,57,58]. It is noteworthy that the positive cluster ions with ammonia [M + NH_4_]^+^, as well as ions formed by elimination of neutral molecules with compositions [M + H-H_2_O]^+^, [M + H-NO_2_]^+^, and [M + H-CO_2_]^+^, were easily found in the positive ESI-MS full scan spectra of AAs. Additionally, the *O*-glucoside derivatives formed ions [M + H-Hex-H_2_O]^+^ and [M + H-Hex-NO_2_]^+^ due to the combined loss of the hexose moiety (162 Da) and the loss of the H_2_O (18 Da) or nitro group (46 Da), respectively. Furthermore, the negative ESI-MS full-scan spectra and MS^2^ fragmentation patterns of the defined compounds were studied and compared with the reference data (Table S1). Consequently, four aristolochic acid analogs were identified: AA-IIIa (4), AA-IVa/b (5), AA-IIIa *O*-glucoside (AA-IIIa-G) (1), and AA-IVa/b *O*-glucoside (AA-IVa/b-G) (3). The chemical structures of the detected compounds are provided in Figure 2.

The accumulation of total AAs in the AC and AB transgenic cell lines was 7–8-fold higher than that of the control A1 calli, but was 1.5–1.6 times less than that in the stems of *A. manshuriensis* (Table 2). While the effects of *rolC* and *rolB* were similar, they varied among different AA derivatives. The most significant increase (12–40 fold) was observed for AA-IIIa, AA-IVa/b, and AA-IIIa-G, whereas moderate effects (6–8 fold) were detected for AA-II and AA-IVa/b-G. In contrast, the content of AA-I was found to be reduced by 5.5 times. The content of magnoflorine was found to be maximum in *rolC*- and *rolB*-transformed hairy roots, exceeding its values in the A1 callus line by 5.8 and 2.8 times, respectively. Consequently, its production in four-week-old AC and AB lines reached 128.7 and 158.7 mg/L, respectively. Principal component analysis (PCA) revealed two significant components, where PC1 accounted for 87.81% of the variance, and PC2 accounted for 9.79%. The loading plot of PC1 was dominated by magnoflorine (Figure S3). At the same time, PC2 was mainly contributed by AA-I, AA-II, AA-IIIa-G, and AA-IVa/b-G, with other metabolites, including magnoflorine, having negative loading values. The samples were divided into four distinct zones based on PC1 and PC2 values (Figure S2), proving that each displayed substantial metabolic discrimination. The A1 and AC cell cultures exhibited the most significant metabolite dissimilarity, suggesting a substantial variance between these groups. Notably, the PCA conducted on the LC-MS and ^1^H NMR data of phytochemicals in 43 *Aristolochia* species indicated that magnoflorine was responsible for the positive PC1 values of the samples [57]. The investigation further demonstrated substantial diversity within discrete *A. manshuriensis* samples, which implies that numerous factors can influence the plant’s phytochemistry [57].

It is well known that *rol* genes considerably alter the biosynthesis of secondary metabolites of the plant. For instance, they increase the content of alkaloids in *Vinca minor* and *Catharanthus roseus* [59,60], flavonoids in *Artemisia annua* [61], anthraquinones in *Rubia cordifolia* [55], and ginsenosides in *Panax ginseng* [37]. Although the *rolB* gene is known for its significant role in enhancing the secondary metabolism of transformed cells [62], its impact on the *A. manshuriensis* hairy roots was not observed to be as prominent as for the *rolC* gene. However, due to the excellent growth characteristics of the *rolB*-transformed hairy roots, the final magnoflorine yield reached the highest level reported in cell culture. Although magnoflorine is a naturally occurring alkaloid, its content in medicinal plants is typically less than 1 mg/g [20]. The yield of magnoflorine has been observed to be in the range of 0.17–0.27 mg/g in different tissues of *A. fangchi* [63]. Likewise, different tissues of *Epimedium alpinum* have been reported to accumulate 0.2–1.0 mg/g of magnoflorine [64]. In the bark of *Ptychopetalum olacoides*, magnoflorine was identified as a major compound, with a recorded concentration of up to 2.56 mg/g [65]. Previous attempts to produce magnoflorine in callus cultures of *Papaver somniferum* and *Stephania glabra* as alternative methods were insufficient in achieving maximum content [66,67]. A combination of transgenic *Escherichia coli* and *Saccharomyces cerevisiae* cells produced only 8.3 mg/L of culture medium [68]. Therefore, transformed hairy root cultures *of A. manshuriensis* can be deemed a promising and novel source of magnoflorine.

To safely use *A. manshuriensis* cell cultures, new strategies for metabolic engineering are needed to reduce AA contents while increasing the concentration of valuable phytochemicals like magnoflorine. A fascinating finding was that the *A. manshuriensis* cell suspension culture, which was transformed with the T-DNA from the wild-type *A. tumefaciens*, exhibited a loss of ability to produce AAs [31]. While the transformation of *A. manshuriensis* with *rolC* and *rolB* genes from *A. rhizogenes* T-DNA did not result in the complete elimination of all AAs, there was a significant reduction in the content of AA-I, which is primarily responsible for the nephrotoxic and carcinogenic effects of *Aristolochia* species [57].

The inactivation of key enzyme genes in the AAs biosynthetic pathway could open up a new possibility for using various AAs-containing plant sources. Developing novel techniques for CRISPR/Cas9-mediated modification of secondary metabolism in plants, calli, and hairy roots with improved traits has emerged as a promising research area [69,70,71,72,73]. Recent study has identified a total of 29 genes that are potentially involved in AA biosynthesis in the *A. contorta* genome [15]. Further investigation of these genes will enable the identification of suitable candidates for targeted manipulations in *Aristolochia* hairy roots.

### 2.3. Free Radical Scavenging Activity of A. manshuriensis Cell Cultures Extracts

DPPH assay evaluated the radical scavenging activity of *A. manshuriensis* plant and cell culture extracts. The results show that AC and AB hairy roots had an almost 2.8- and 2.9-fold higher antioxidant capacity than control calli, respectively, and were similar to those in *A. manshuriensis* stems (Table 3).

Antioxidant activities have been previously studied in extracts of *A. indica* [74,75], *A. bracteata* [76], *A. longa* [77], *A. clematitis* [78], and *A. albida* [79]. The results demonstrated that DPPH scavenging activity varied between 20 and 550 µg/mL and that roots have a higher capacity for free radicals than areal parts of the plants. The antioxidant activity of *Aristolochia* plants was primarily due to magnoflorine [80]. In particular, magnoflorine demonstrated remarkable antioxidant activity, as a dose of 50 μg/mL was found to scavenge approximately 70.8% of all the free radicals [81].

There has been limited research on the antioxidant properties of cell cultures derived from *Aristolochia* plants. Despite the accumulation of significant levels of magnoflorine in A1 calli, their extract demonstrated DPPH scavenging activity which was 37.9% of that found in the stem. This study is the first to demonstrate that *rolC* and *rolB* genes can effectively enhance the antioxidant activity of hairy roots to levels comparable to those found in wild *A. manshuriensis* liana stems, likely due to the higher concentration of magnoflorine. It is important to highlight that the DPPH-based measurements of antioxidant capacity should be utilized cautiously because they might not accurately represent the activity of extracts in vivo. Further investigations are necessary to fully elucidate the underlying mechanism of this phenomenon and explore its potential applications in fields such as medicine.

### 2.4. Cytotoxic Effect of Constituents from A. manshuriensis Cell Cultures

**Figure 3:**
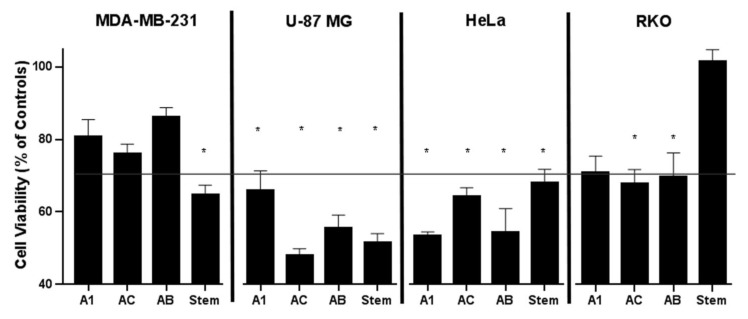
Cell viability of MDA-MB-231, U-87 MG, HeLa CCL-2, and RKO cell lines in response to A1, AC, AB, and liana stems extracts, evaluated by MTT assay. Data are presented as the mean values ± standard errors. All data, with the exception of stem extract against RKO, are significantly different from the corresponding controls (set as 100%) at *p* < 0.01 (Student’s *t*-test). Asterisk designates viability inhibition greater than 30%.

The cytotoxicity of A1, AC, AB, and stem extracts was examined by MTT assay on four cell lines: triple-negative breast cancer cells (MDA-MB-231), human glioblastoma cells (U-87 MG), cervical cancer cells (HeLa CCL-2), and human colon carcinoma cells (RKOs) (Figure 3). The extract from the A1 callus line reduced the activity of cancer cell lines HeLa CCL-2, U87MG, RKO, and MDA-MB-231 by 47%, 35%, 30%, and 19.2%, respectively. AC extracts reduced the metabolic activity of U-87 MG by 53% and HeLa CCL-2 cells by 36%, as well as RKO by 33% and MDA-MB-231 by 23.9%. AB extracts reduced the activity of HeLa CCL-2, U-87 MG, RKO, and MDA-MB-231 by 46%, 45%, 31%, and 13.8%, respectively. Stem extracts showed inhibitory effects on U-87 MG, MDA-MB-231, and HeLa CCL-2 cell growth at 48.4%, 35.4%, and 32.1%, respectively, while no cytotoxicity was observed with respect to RKO (Figure 3). In general, our findings indicate that extracts from hairy roots exhibited significant cytotoxic effects that differed across various cancer cell lines, implying that the impact was specific to each cell type.

The inhibitory properties of *Aristolochia* extracts may be attributed to the presence of magnoflorine [80]. Magnoflorine has been found to exhibit cytotoxic effects against several cancer cell lines, including those of brain, gastric, breast, and lung tumors, as well as hepatocellular carcinoma, among others [20,82,83,84,85,86]. The anti-tumor effects of magnoflorine have been linked to the induction of reactive oxygen species (ROS)-mediated apoptosis and autophagy through the AKT/mTOR and p38 signaling pathways [85,86]. However, some studies have reported that magnoflorine does not show cytotoxic effects against particular human cancer cell lines, such as KB, SiHa, A549, HaCaT, SK-OV-3, SK-MEL-2, XF498, HT-29, and HCT15 [81]. Moreover, magnoflorine is non-toxic to human embryonic kidney cells (HEK293) and normal gastric cells [81,85].

### 2.5. The Influence of A. manshuriensis Cell Cultures Extract on Cell Cycle of Cancer Cells

**Figure 4:**
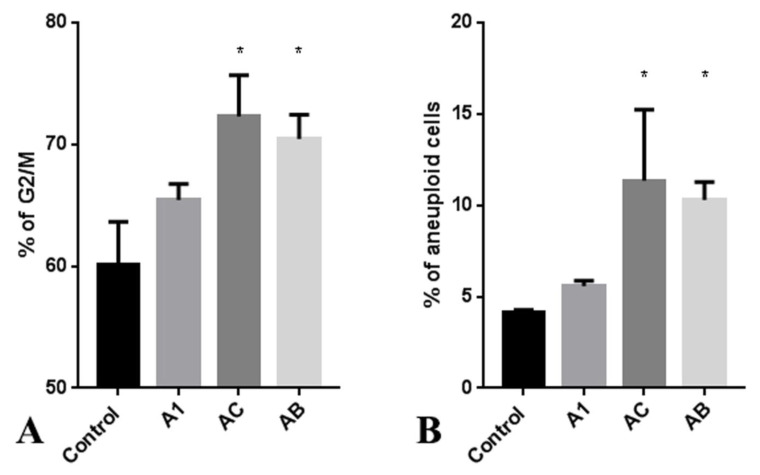
Cell cycle analysis (**A**) and aneuploidy percentage (**B**) of RKO cancer cells after treatment with A1, AC, and AB extracts. Data are presented as the mean values ± standard errors, * *p* < 0.05 (Student’s *t*-test).

Upon observing the antiproliferative effects of *A. manshuriensis* cell culture extracts, cell cycle analysis was performed on the RKO cell line. Extracts obtained from AC and AB hairy roots induced a significant increase in the accumulation of G2/M phase cells compared to control calli (Figure 4A and Figure S4). Additionally, the treatment of RKO cancer cells with extracts from hairy roots resulted in a more than 10% increase in aneuploidy (Figure 4B and Figure S5). These findings suggest that the constituents of AC and AB extracts may trigger chromosome instability, leading to cell death and explaining the observed anti-proliferative effects of *A. manshuriensis* extracts via DNA damage. Previous studies have reported that magnoflorine induces ROS-induced apoptosis in cancer cells [85,86]. Other studies have demonstrated that ROS-induced apoptosis can be mediated by DNA double-strand breaks (DSBs) [87]. G2/M checkpoint arrest is a well-known mechanism that protects continuous cell cycles by preventing inaccurate chromosome segregation and leading to programmed cell death before critical genome instability can occur.

Studies have demonstrated that *A. debilis* extract induces sub-G1 arrest and apoptosis in HT-29 cells through changes in mitochondria-dependent apoptosis markers and the accumulation of ROS [88]. Similarly, magnoflorine treatment of gastric cancer cells has been shown to cause cell cycle arrest at the S/G2 phase via activation of ROS-dependent downstream signaling [84,85,86]. Another study revealed that extracts from *A. clematitis*, *A. elegans*, *A. acuminate*, and *A. guentheri* induced G2/M phase arrest and apoptosis in human kidney cells (HK-2) [57]. The observed levels of cytotoxicity, micronuclei induction, G2/M arrest, and apoptosis were found to be associated with aristolactam BI, AA-D, AA-IIIa, and AA-IIIa-G. Interestingly, the latter two metabolites were significantly upregulated in AC and AB hairy roots (Table 2). Surprisingly, the content of AA-I and AA-II, which are highly toxic agents found in *Aristolochia* species, did not correlate with the effects above [57]. These compounds were the most common aristolochic acid derivatives in A1 and stem, comprising 41–44% of total AAs. However, they only made up 3–5% of *rol*-transformed hairy roots of *A. manshuriensis* (Table 2). Thus, *rolB* and *rolC* genes induced alterations in the secondary metabolism of the hairy roots of *A. manshuriensis* that prevail in reducing toxicity and increasing valuable compounds. However, further research is still needed to validate the safety of obtained hairy roots, such as acute and long-term toxicology assessments on animals.

## 3. Materials and Methods

### 3.1. Plant Material and Tissue Cultures

In this study, we used parts of a wild-growing *Aristolochia manshuriensis* liana that had been collected in Nadegdinsky District (Nadegdinsk, Primorsky Krai, Russia). The control, untransformed callus culture (A1) was derived from 1-year-old *A. manshuriensis* stems as described in [34] and cultivated using W medium [89] supplemented with 1 mg/L 6-benzylaminopurine and 1 mg/L indole-3-acetic acid under darkness at 25 °C with a 30-day subculture cycle. During the period of observation (more than 10 years), A1 did not show any rhizogenic effects.

Petiole, leaf, and stem explants of 3-week-old clonally cultivated *A. manshuriensis* plantlets were inoculated with the GV3101 strains of *Agrobacterium tumefaciens* [90] carrying pPCV002-A, pPCV002-CaMVBT, and pPCV002-CaMVC binary vectors [48] (Figure S6). In these constructs, the *rolA* gene is driven by its native promoter, while the *rolB* and *rolC* genes are under the control of the cauliflower mosaic virus (CaMV) 35S promoter. The T-DNA region also contains the gene for kanamycin resistance (*nptII*) under eukaryotic control sequences. As a control, we used *A. thumefaciens* carrying an empty pPCV002 vector (Figure S5). The optimized transformation protocol was previously described in detail [37]. In brief, the sterilized explants were cut into small pieces with the scalpel and dipped into the *A. thumefaciens* suspension in MES buffer (10 mM MES; pH, 5.6; 10 mM MgCl2; 100 μM acetosyringone) with OD_600_ = 0.5 for 10 min. After incubation, inoculated explants were blotted dry and then kept for 2 days on agar-solidified W medium containing 100 µM acetosyringone at 25 °C in the dark. Thereafter, explants were transferred to fresh medium supplemented with 250 mg/L cefotaxim and 50 mg/L kanamycin to produce lines of primary kanamycin-resistant tumors. After 3 weeks, *rolC*- and *rolB*-transformed primary tumors were able to spontaneously form adventitious roots. Root tips, isolated from adventitious roots were transferred into liquid W medium supplemented with 0.5 mg/L indole-3-butyric acid to further promote root growth. These cultures, designated as AC and AB, were cultivated in the dark at 25 °C in 500 mL Erlenmeyer flasks in an orbital shaker (100 rpm; 20 mm amplitude) and subcultured at 28-day intervals.

### 3.2. DNA Isolation and PCR Analysis

The isolation of genomic DNA from dry callus tissues was carried out with a CTAB-based method [91]. DNA quality was examined by electrophoresis on a 0.8% agarose gel. Spectrophotometric analysis using a BioSpec-nano spectrophotometer (Shimadzu, Kyoto, Japan) was employed to estimate the quantity and purity of extracted DNA.

We first aimed to amplify *A. manshuriensis* DNA sequences corresponding to the housekeeping actin gene. For this aim, PCRs were performed in 25 µL reaction volumes containing 50 ng of template DNA, 1× PCR buffer with 2.5 mM MgCl_2_, 200 μM of each deoxynucleotide, 1 μM of the primer pair (Table S2), and 1 U Taq polymerase (Evrogen, Russia). PCRs were performed in an iCycler (Bio-Rad Laboratories), and cycle conditions were as follows: 96 °C for 10 s, 50 °C for 30 s, and 72 °C for 1 min. DNA fragments of predicted lengths were obtained, isolated from agarose gels with a Glass Milk Kit (Sileks, Russia), and sequenced as described earlier [55] at the Instrumental Center of Biotechnology and Gene Engineering of FSCEATB FEB RAS using an ABI 3500 Genetic Analyzer (Applied Biosystems, Foster City, CA, USA). This allowed identification of two actin isoforms, namely, *AmACT1* (GenBank accession no.: OQ676410) and *AmACT2a* (GenBank accession no.: OQ676411), of which the latter sequence contains an 88 bp intron. To verify successful T-DNA integration, PCRs were performed with the above conditions and primer sets specific to the *rolB*, *rolC*, *nptII*, and *AmACT1* genes (Table S2).

### 3.3. High-Performance Liquid Chromatography (HPLC) Analysis

For secondary metabolite analysis, *A. manshuriensis* calli were dried under hot air for 20 h and ground using a mortar and pestle.

#### 3.3.1. Chemicals

Analytical standards (magnoflorine, AA-I, and AA-II) were obtained from Sigma-Aldrich (St. Louis, MO, USA). Milli-Q water (Millipore, Bedford, MA, USA) was used for preparing standard solutions, extraction buffers, and eluents. Formic and acetic acids were of high-performance liquid chromatography (HPLC)-grade and obtained from Merck (Darmstadt, Germany). HPLC-grade acetonitrile (ACN) was obtained from PanReac AppliChem (Darmstadt, Germany). All the other chemicals were of analytical-grade.

#### 3.3.2. Sample Preparation and HPLC Assays

Dried and ground samples (50 mg) were supplemented with 1 mL 70% ethanol, homogenized, sonicated for 30 min at 40 °C, and centrifuged at 15,000× *g* for 15 min at 4 °C. The supernatants were collected, filtered (0.45 μm nylon membrane, Millipore, Bedford, MA, USA), and analyzed by LC-UV-MS(MS^2^) as we described previously [92,93] with a few additions. Briefly, the HPLC-UV-MS studies were performed with an Agilent 1260 Infinity analytical HPLC system (Agilent Technologies, Santa Clara, CA, USA) equipped with a photodiode array detector, an analytical Zorbax C18 column (150 mm, 2.1 mm i.d., 3.5-μm part size, Agilent Technologies, USA), and interfaced with an ion trap mass spectrometer Bruker HCT Ultra PTM Discovery System (Bruker Daltonik GmbH, Bremen, Germany). High-resolution mass spectrometry (HRMS) was performed using a Shimadzu LCMS-IT-TOF instrument (Shimadzu, Kyoto, Japan) equipped with an ion trap time-of-flight mass spectrometer operating under electrospray ionization (ESI) conditions. All chromatographic and mass spectrometric conditions were the same [92]; detailed information is presented in Table S1 and Supplementary HPLC-UV and HRMS data. Chromatograms for quantification were recorded at 237 nm (for magnoflorine) and 254 nm (for AAs). Quantification of magnoflorine, AA-I, and AA-II was performed by corresponding peak areas using the absolute calibration method with commercially available standards. The amounts of AA-IIIa, AA-IVa/b, AA-IIIa-G, and AA-IVa/b-G, for which standards were not available, were calculated using the calibration curve of AA-I and corrected according to their molecular weight.

### 3.4. Antioxidant Activities of Extracts

The antioxidant activity of the extracts was determined using a 2,2-diphenyl-1-picrylhydrazyl (DPPH) free radical scavenging photometric assay [93]. A solution of 0.3 mM DPPH in ethanol was prepared, and 149 µL of this solution was mixed with 1 µL of extracts in ethanol at different concentrations (5–500 µg/mL). For comparison, ascorbic acid (vitamin C) was used. After incubation at room temperature, the absorbance was measured at 517 nm using a Benchmark Plus microplate spectrophotometer (Bio-Rad, Hercules, CA, USA) in 3 technical replicates and converted into percentage inhibition (%*inh*) using the following formula:

(1) $$\%inh=\frac{\left(\left({A_{B}-A}_{S}\right)\times 100\%\right)}{\left(A_{B}\right)},$$

where *A_B_* is the absorbance of the negative control (equal amount of DPPH solution), and *A_S_* is the absorbance of the test sample. A similar amount of extract in ethanol was used as a blank. The IC_50_ values were calculated by using linear regression of plots, where the abscissa represented the concentration of tested extracts and the ordinate the average percent of antioxidant activity from three separate tests.

### 3.5. Anticancer Activities of Extracts

Extracts were tested on four cell lines using the MTT assay: triple-negative breast cancer cells (MDA-MB-231), human glioblastoma cells (U-87 MG), cervical cancer cells (HeLa CCL-2), human colon carcinoma cells (RKOs), all received from ATCC. Above cell cultures were cultivated in the Laboratory of Biomedical Cell Technologies at the Institute of Life Sciences and Biomedicine of Far Eastern Federal University.

Standard cell models were seeded onto a 96-well plate at a concentration of 5 × 10^3^ cells per well in the appropriate culture medium. Since the extracts were dissolved in methanol (MeOH), we used 0.5% MeOH as the negative control added to the medium with cells (this concentration of the solvent is considered non-toxic). For convenience in testing and adding extracts during the experiment, we prepared master mixes with both the extracts and MeOH for the negative control at a concentration of 0.5%. At 24 h after seeding, the extracts from the prepared master mixes and MeOH as the control were added to the 96-well plate at a volume of 120 μL per well. The cells with added extracts were incubated for 72 h at 37 °C, 5% CO_2_, and 80% relative humidity. After incubation, cytotoxicity analysis was conducted using the MTT assay as previously described [94].

### 3.6. Cell Cycle Analysis

Cells (100 × 10^3^ cells/well) were seeded in 6-well culture plates and treated for 5 days with different concentrations of extracts (up to a final methanol concentration of 0.5%) in three independent replicates. Pure methanol was added as a control up to a final concentration of 0.5%. After the incubation period, the cells were detached with a 0.5% trypsin solution and fixed with 4% paraformaldehyde. After a series of washes, the suspension was stained with the intercalating dye Hoechst 33342 at a concentration of 10 μg/mL for 20 min, washed, and analyzed using the BD FACS MoFlo Astrios cell sorter (BD Biosciences, Indianapolis, IN, USA) with a 355 nm ultraviolet laser. Emission was recorded at 488 nm. Results were analyzed using Kaluza 2.1 software with the cell cycle analysis function based on the Michael H. Fox algorithm.

### 3.7. Statistical Analysis

All values were expressed as the mean ± SE. For statistical evaluation, Student’s *t*-test was used to compare the two independent groups. For comparison among multiple data sets, analysis of variance (ANOVA) followed by multiple comparison procedures was employed. Fisher’s protected least significant difference (PLSD) post hoc test was employed for the intergroup comparison. PAST 4.03 software was used for principal component analysis (PCA) of secondary metabolites detected using HPLC-UV-MS [95].

## 4. Conclusions

This study reports the establishment of hairy root cultures of the rare and valuable medicinal plant *A. manshuriensis* through the genetic transfer of individual *rolC* and *rolB* genes. The results indicate that both *rolC* and *rolB* genes effectively induced the formation of roots on petiole and stem explants, with *rolB* having a more pronounced effect. The findings highlight the importance of selecting the appropriate explant and *rol* gene to induce hairy root syndrome in *A. manshuriensis*. Furthermore, it was found that both *rolC* and *rolB* genes led to a significant increase in AAs and magnoflorine in transformed *A. manshuriensis* cells, but to varying degrees. In the AC and AB cell lines, the accumulation of AA-I decreased considerably, while the contents of AA-IIIa, AA-IVa/b, and AA-IIIa-G increased up to 40 fold. The *rolC* gene had the most significant effect on the upregulation of magnoflorine quantity, with the AC cell line reaching 5.72 mg/g of dry weight. In addition, the results show that the hairy root extracts of *A. manshuriensis* exhibited significant cytotoxic effects on human glioblastoma cells (U-87 MG), cervical cancer cells (HeLa CCL-2), and colon carcinoma (RKO) cells. However, when tested on triple-negative breast cancer cells, the extracts did not show significant activity (MDA-MB-231). Further research is needed to explore the potential of *A. manshuriensis* hairy roots as a source of anti-cancer agents and to elucidate the underlying mechanisms of action.

## Figures and Tables

**Table 1:** The effectiveness of hairy root initiation from different explants, %.

	Stem	Petiole	Leaf
Control	-	-	-
*rolA*	-	-	-
*rolC*	10.8 ± 1.9	5.9 ± 0.6 **	-
*rolB*	20.1 ± 2.3	8.3 ± 0.9 **	-

Explants were considered transformed if they displayed at least one sign of hairy roots. The data presented are mean values ± standard errors. Data are presented as the mean values ± standard errors, ** *p* < 0.01 (Student’s *t*-test).

**Table 2:** Aristolochic acids and magnoflorine content (mg/g of dry weight) in cell cultures and stems of *A. manshuriensis* measured by using HPLC-UV-MS.

	A1	AC	AB	Stem
Magnoflorine	0.99 ± 0.102 ^D^	5.72 ± 0.686 ^A^	2.76 ± 0.304 ^C^	3.39 ± 0.475 ^B^
AA-I	0.11 ± 0.010 ^B^	0.02 ± 0.002 ^C^	0.02 ± 0.002 ^C^	1.23 ± 0.139 ^A^
AA-II	0.01 ± 0.001 ^C^	0.08 ± 0.010 ^B^	0.06 ± 0.007 ^B^	0.34 ± 0.027 ^A^
AA-IIIa	ND	0.12 ± 0.016 ^B^	0.22 ± 0.033 ^A^	0.04 ± 0.006 ^C^
AA-IVa/b	0.01 ± 0.001 ^C^	0.15 ± 0.020 ^B^	0.29 ± 0.034 ^A^	ND
AA-IIIa-G	0.02 ± 0.003 ^B^	0.77 ± 0.098 ^A^	0.80 ± 0.119 ^A^	0.82 ± 0.109 ^A^
AA-Iva/b-G	0.14 ± 0.019 ^C^	0.99 ± 0.133 ^AB^	0.94 ± 0.136 ^B^	1.18 ± 0.154 ^A^
Sum of Aas	0.29 ± 0.037 ^C^	2.13 ± 0.280 ^B^	2.32 ± 0.266 ^B^	3.61 ± 0.513 ^A^

Data are presented as the mean values ± standard errors. The same superscript letters in the rows denote no significant differences at the 0.05 level, Fisher’s LSD test. ND—not detected.

**Table 3:** DPPH radical scavenging activity of cell cultures and stems of *A. manshuriensis*.

Sample	IC_50_ (µg/mL)
Vitamin C	276 ± 2.29 ^A^
A1	116 ± 4.71 ^B^
AC	41 ± 1.49 ^C^
AB	40 ± 2.41 ^C^
Stem	44 ± 1.54 ^C^

Data are presented as the mean values ± standard errors. The same superscript letters in the rows denote no significant differences at the 0.05 level, Fisher’s LSD.

## Data Availability

All data are contained within the article and Supplementary Materials. The datasets generated during and/or analyzed during the current study are available from the corresponding author upon reasonable request.

## Supplementary Materials

The supporting information can be downloaded at: https://www.mdpi.com/article/10.3390/ijms241411240/s1.
