# Supplementary material for: Enhanced Production of Nitrogenated Metabolites with Anticancer Potential in Aristolochia manshuriensis Hairy Root Cultures
Source: Int J Mol Sci. 2023 Jul 8;24(14):11240. doi: 10.3390/ijms241411240 (PMC10379662; doi:10.3390/ijms241411240)
Supplement: Supplementary file 1 [file ijms-24-11240-s001.zip › Supplementary Materials-rev.pdf]

Supplementary Materials

Enhanced production of nitrogenated metabolites with antioxidant and anticancer potential in *Aristolochia manshuriensis* hairy root cultures

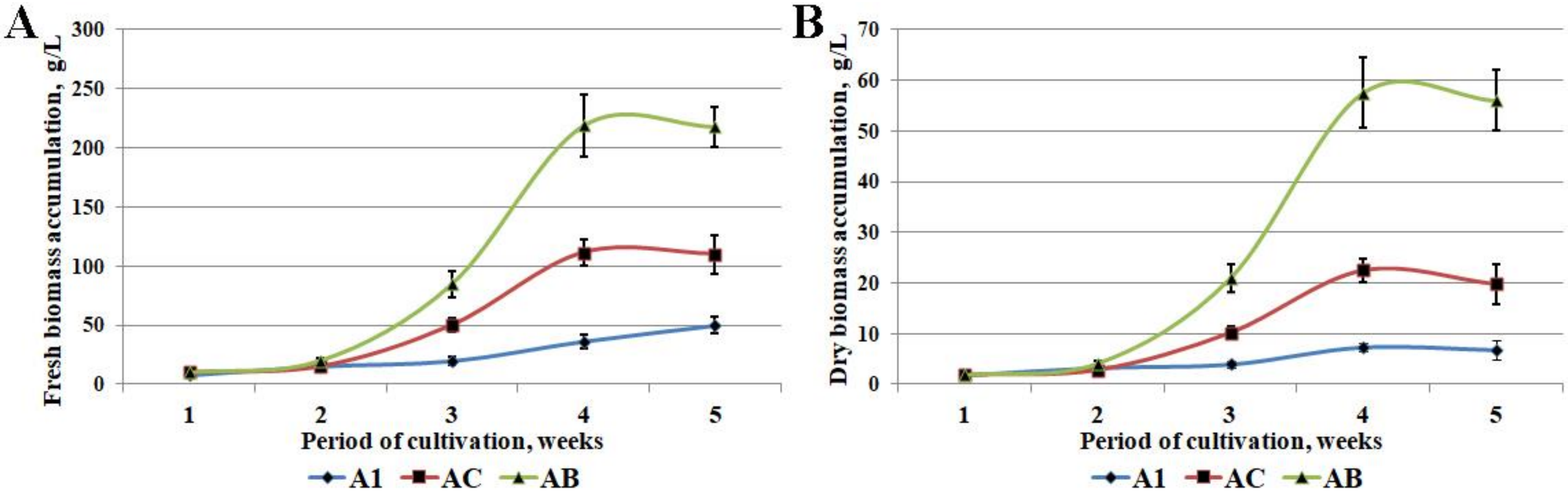

Figure S1. Growth profile of *A. manshuriensis* cell cultures at the specified culture times (week) as measured by fresh (**A**) and dry (**B**) weight (g/L). Data are presented as the mean ± standard errors from three biological replicates.

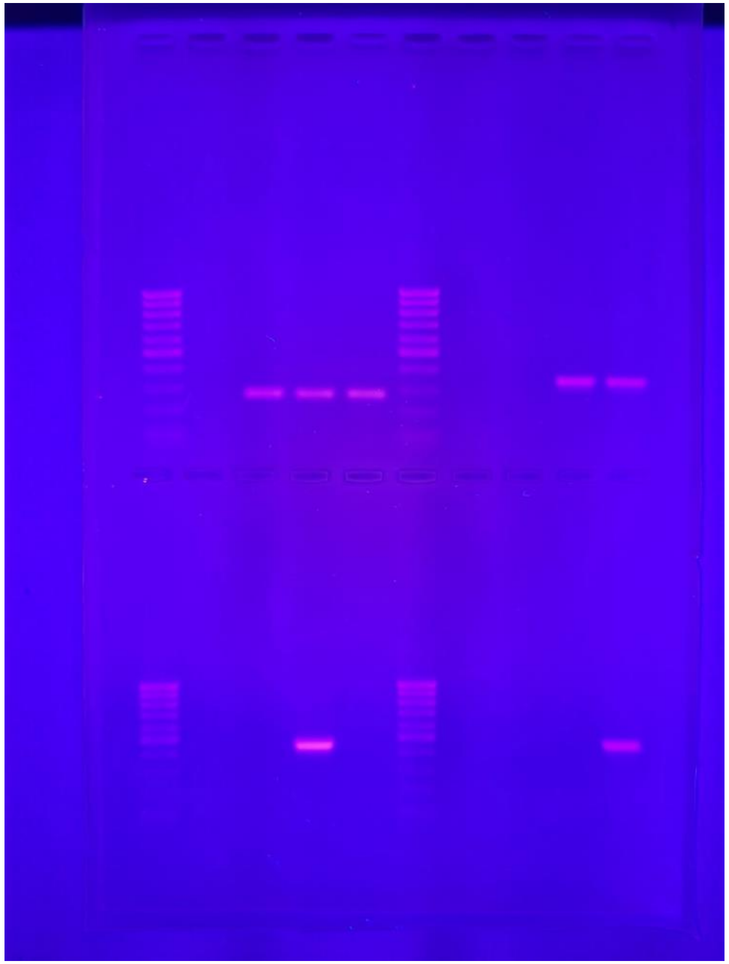

Figure S2. Unprocessed and uncropped image of the agarose gel electrophoresis reported in Figure 1.

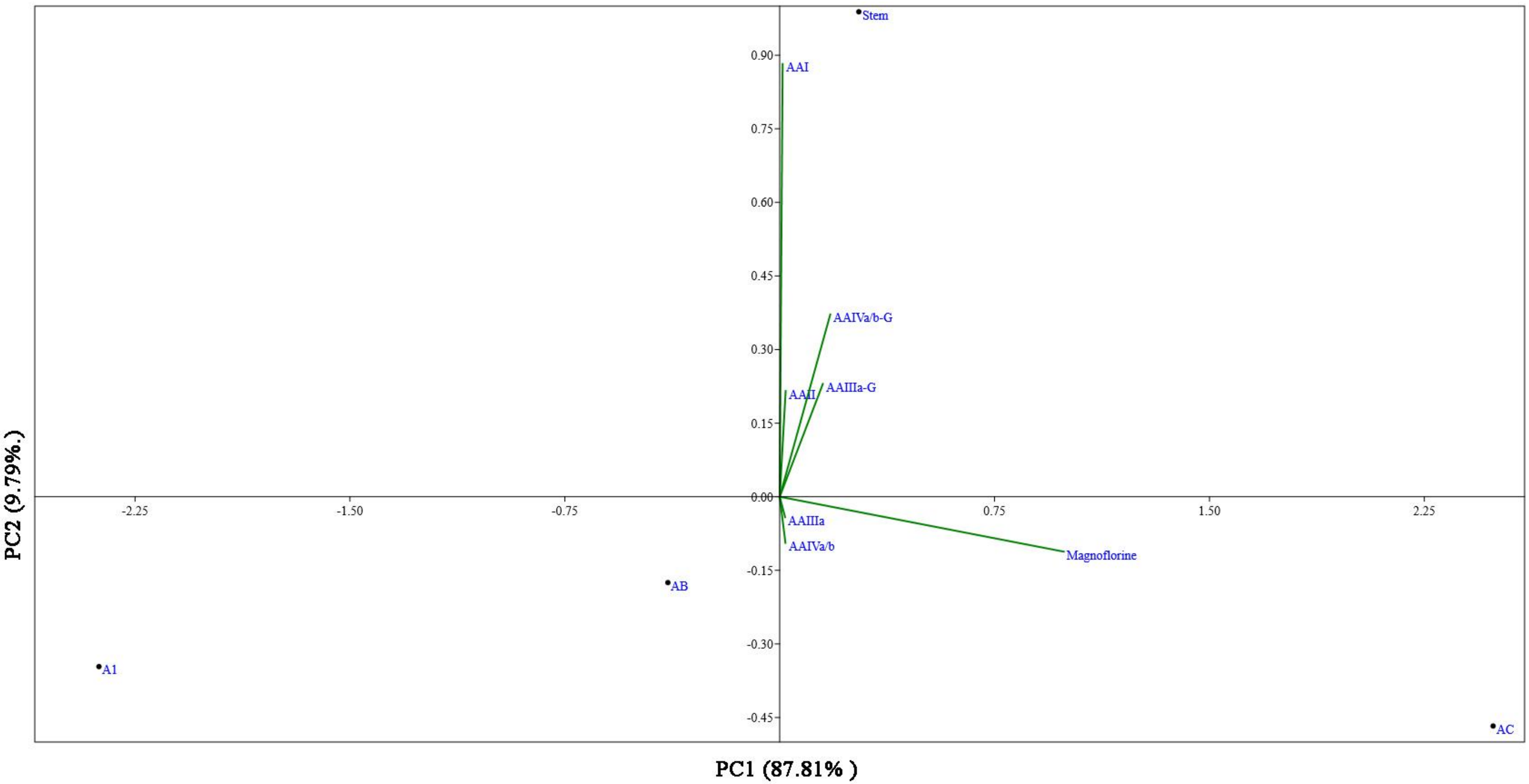

Figure S3. Principal component analysis (PCA) of HPLC-UV data from samples of *A. manshuriensis*.

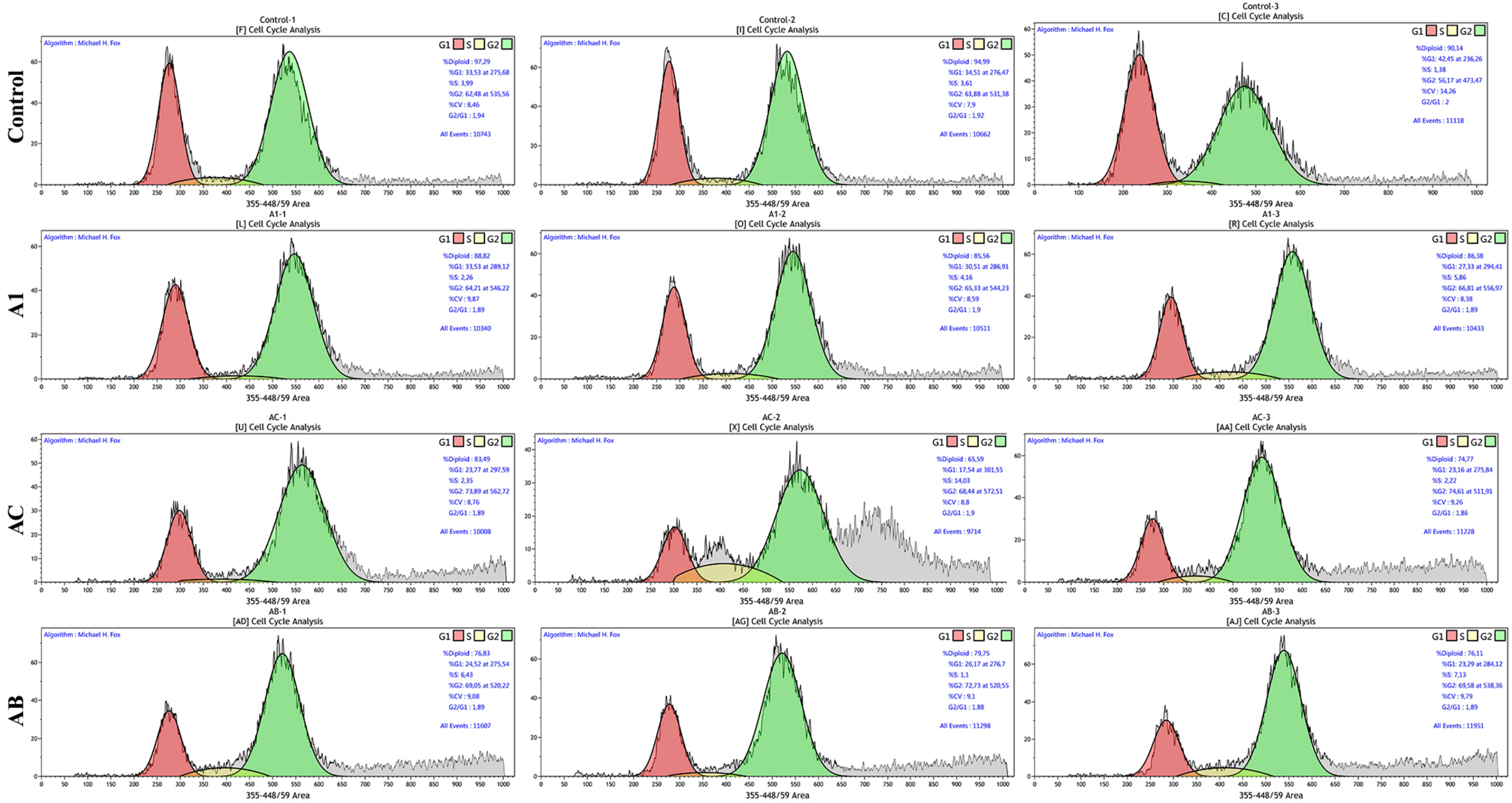

Figure S4. Cell cycle analysis of RKO cell culture using the Michael H. Fox algorithm with Kaluza 2.1 software after treatment with methanol (Control), A1, AC, and AB extracts.

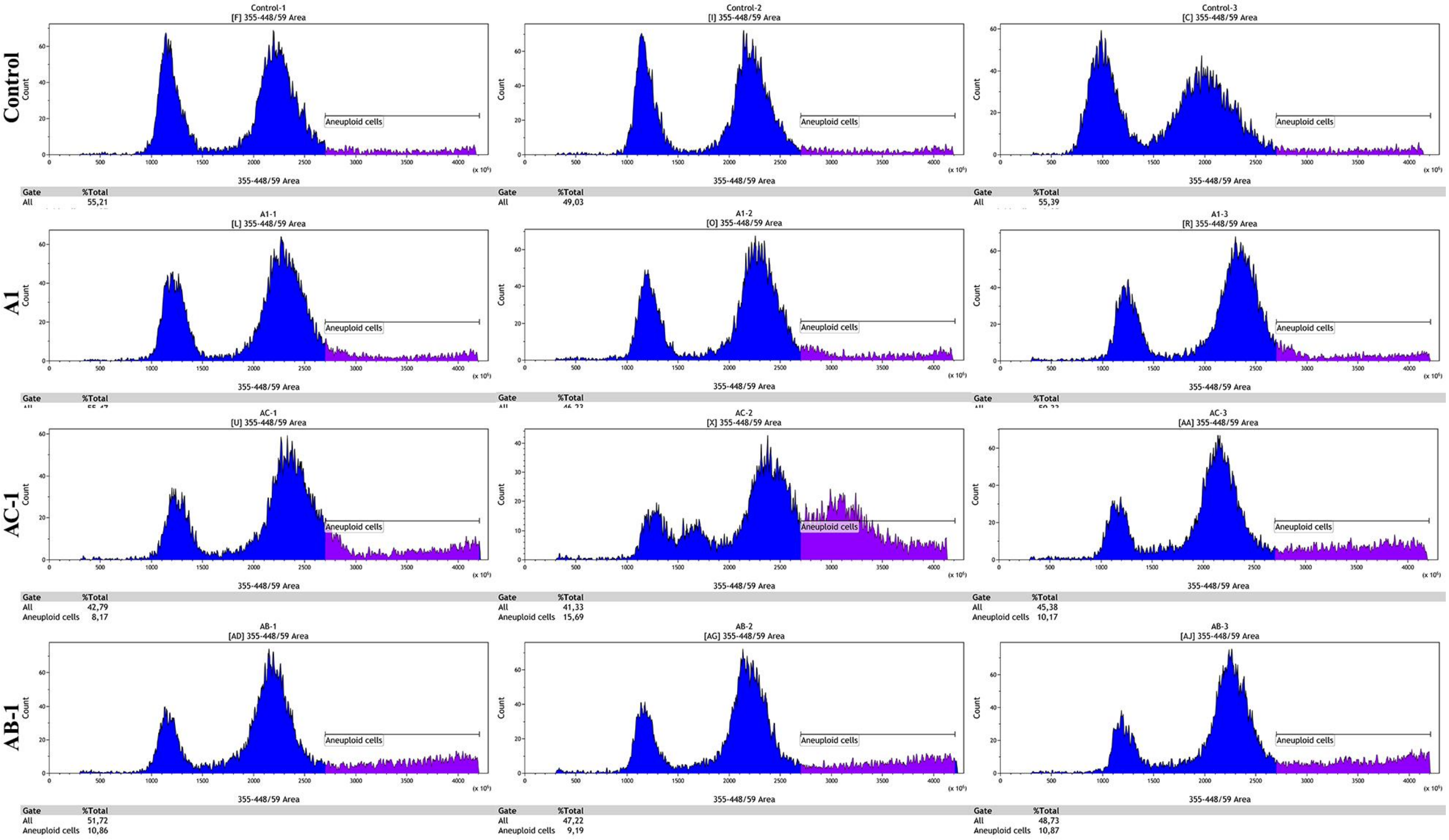

Figure S5. DNA ploidy analysis of RKO cell culture using Kaluza 2.1 software after treatment with methanol (Control), A1, AC, and AB extracts.

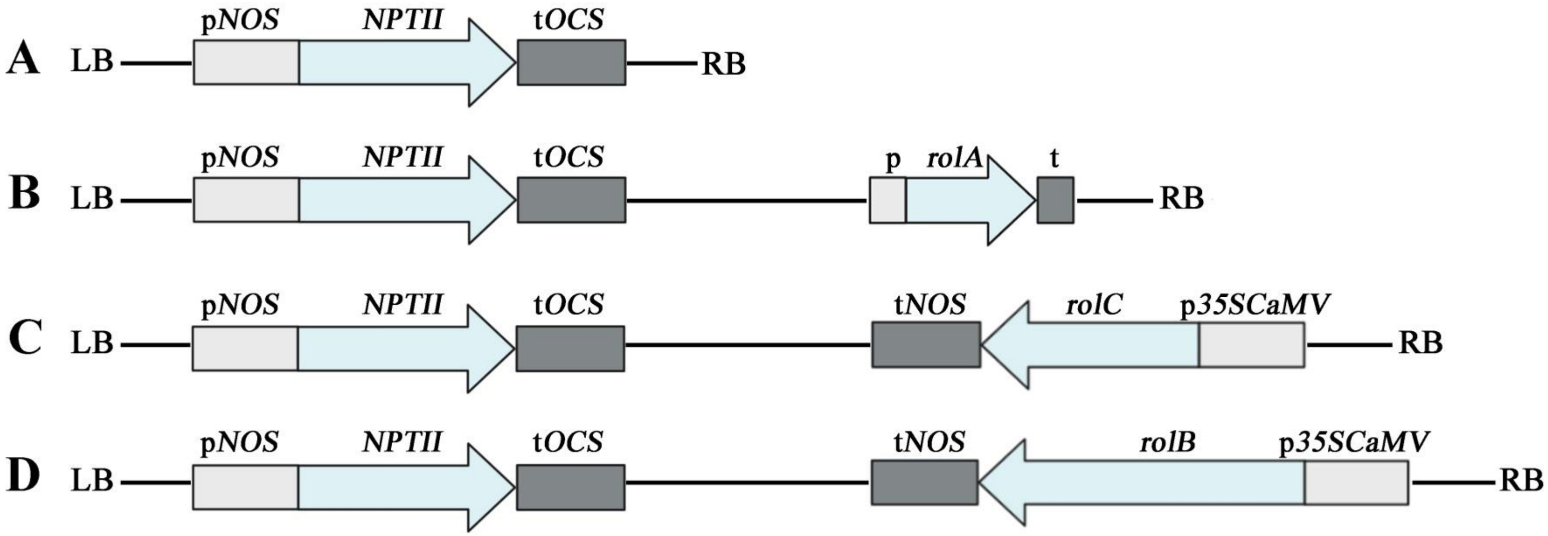

Figure S6. Schematic representation of the T-DNA region from the empty pPCV002 (**A**), pPCV002-A (**B**), pPCV002-CaMVC (**C**), and pPCV002-CaMVBT (**D**) binary vectors.

Table S1. List of phenanthroic acid derivatives identified in cell cultures and stems of *A. manshuriensis* measured by using HPLC-MS.

Peak no.	Rt (min)	UVmax (nm)	Detected ions composition	Detected ions (m/z)	Diff (ppm)	Diff (mDa)	Molecular Formula	MS2 fragmentation, main diagnostic ions (m/z)	Compound assignment	References
1	13.3	258, 305	$[M+NH_4]+$	507.1257	2.3	1.15	$C_{22}H_{19}NO_{12}$		Aristolochic acid IIIa O-glucoside*	56
		350-380	$[M+H-Hex-H_2O]+$	310.0336	3.3	1.01		266, 208		57
			$[M+H-Hex-NO_2]+$	282.0542	6.8	1.93				58
			[M-H]−	488.0844	1.4	0.95		279		
			[M+HCOO]−	534.0882	1.5	0.73		488		
2	14.2	228, 273, 305	M+	342.1683	4.9	1.69	$C_{20}H_{24}NO_4+$	311, 297, 279, 265	Magnoflorine**	St.
			[M-2H]−	340.1551	1.0	0.33		325		56
			[M-H+HCOO]−	386.1608	0.3	0.11		340		57
3	15.5	255, 325, 402	$[M+NH_4]+$	537.1330	4.1	2.12	$C_{23}H_{21}NO_{13}$	474, 312	Aristolochic acid IVa/IVb *O*-glucoside*	56
			$[M+H-NO_2]+$	474.1169	2.6	1.24		312		58
			$[M+H-Hex-H_2O]+$	340.0432	5.8	1.98		296, 281, 253		
			$[M+H-Hex-NO_2]+$	312.0614	4.6	1.44		297, 269, 267		
			[M-H]−	518.0961	4.0	2.09		309		
			[M+HCOO]−	564.0996	0.2	0.11		518, 309		
4	19.3	257, 304,	$[M+H-H_2O]+$	310.0338	2.6	0.81	$C_{16}H_9NO_7$	266, 238	Aristolochic acid IIIa*	56
		350-380	$[M+H-NO_2]+$	282.0520	1.0	0.28				58
			[M-H]−	326.0294	3.7	1.23		280, 236		
			$[M-H-NO_2]-$	280.0364	4.7	1.32		236		
5	21.6	255, 325, 405	$[M+NH_4]+$	375.0816	1.9	0.69	$C_{17}H_{11}NO_8$	358, 312	Aristolochic acid IVa/IVb*	56
			$[M+H-H_2O]+$	340.0434	5.2	1.78		296, 281		58

			[M+H-NO2]+	312.0618	3.3	1.04		297, 269		
			[M-H]−	356.0399	3.6	1.29		310, 266		
			[M-H-NO2]-	310.0467	5.1	1.59		266		
6	24.5	250, 303, 350-380	[M+NH4]+	329.0760	2.6	0.81	$C_{16}H_9NO_6$	294, 268	Aristolochic acid II**	St.
			[M+Na]+	334.0310	3.9	1.21				56
			[M+H-H2O]+	294.0382	5.1	1.50		279, 250, 222, 192		57
			[M+H-CO2]+	268.0615	4.0	1.07		238		58
			[M-H+HCOONa]−	378.0245	4.1	1.36		266		
			[2M-2H+Na]-	643.0615	1.3	0.86		379, 266		
7	26.4	249, 319, 395	[M+NH4]+	359.0860	4.0	1.38	$C_{17}H_{11}NO_7$	342, 324, 298	Aristolochic acid I**	St.
			[M+Na]+	364.0410	5.2	1.77		318		56
			[M+H-H2O]+	324.0491	3.6	1.16		280, 265, 237, 222		57
			[M+H-CO2]+	298.0698	4.0	1.20		281, 268, 252, 222		58
			[M+H-NO2]+	296.0665	4.8	1.43		281		
			[M-H+HCOONa]−	408.0348	3.0	1.10		296		
			[2M-2H+Na]-	703.0807	1.5	1.07		296		

* The compound was identified by comparison with the literature data.

** The compound was identified by comparison with reference standard (St.).

Table S2. List of primer sequences used in this study.

Gene name<br><br>(GenBank accession No.)	Forward (5′ to 3′)	Reverse (5′ to 3′)
nptII (AY818371)	TGATATTCGGCAAGCAGGCA	TTGTCACTGAAGCGGGAAGG
rolC (K03313)	GAGCGTAAACCCTTGATCGA	CCGATTGCAAACTTGCACTC
rolB (K03313)	TTGTCTATCTTTCTCGCGAG	CTTCAGGTTTACTGCAGCAG
AmAct1 (OQ676410)	AGATTCCGATGCCCTGAGGT	TCCTCCAATCCAAACGCTGT
